# Stretch-Induced Stress Fiber Remodeling and the Activations of JNK and ERK Depend on Mechanical Strain Rate, but Not FAK

**DOI:** 10.1371/journal.pone.0012470

**Published:** 2010-08-30

**Authors:** Hui-Ju Hsu, Chin-Fu Lee, Andrea Locke, Susan Q. Vanderzyl, Roland Kaunas

**Affiliations:** Department of Biomedical Engineering, Texas A&M University, College Station, Texas, United States of America; University Medical Center Utrecht, Netherlands

## Abstract

**Background:**

Cells within tissues are subjected to mechanical forces caused by extracellular matrix deformation. Cells sense and dynamically respond to stretching of the matrix by reorienting their actin stress fibers and by activating intracellular signaling proteins, including focal adhesion kinase (FAK) and the mitogen-activated proteins kinases (MAPKs). Theoretical analyses predict that stress fibers can relax perturbations in tension depending on the rate of matrix strain. Thus, we hypothesized stress fiber organization and MAPK activities are altered to an extent dependent on stretch frequency.

**Principal Findings:**

Bovine aortic endothelial cells and human osteosarcoma cells expressing GFP-actin were cultured on elastic membranes and subjected to various patterns of stretch. Cyclic stretching resulted in strain rate-dependent increases in stress fiber alignment, cell retraction, and the phosphorylation of the MAPKs JNK, ERK and p38. Transient step changes in strain rate caused proportional transient changes in the levels of JNK and ERK phosphorylations without affecting stress fiber organization. Disrupting stress fiber contractile function with cytochalasin D or Y27632 decreased the levels of JNK and ERK phosphorylation. Previous studies indicate that FAK is required for stretch-induced cell alignment and MAPK activations. However, cyclic uniaxial stretching induced stress fiber alignment and the phosphorylation of JNK, ERK and p38 to comparable levels in FAK-null and FAK-expressing mouse embryonic fibroblasts.

**Conclusions:**

These results indicate that cyclic stretch-induced stress fiber alignment, cell retraction, and MAPK activations occur as a consequence of perturbations in fiber strain. These findings thus shed new light into the roles of stress fiber relaxation and reorganization in maintenance of tensional homeostasis in a dynamic mechanical environment.

## Introduction

Cytoskeletal tension enables cells to adhere, spread, contract, and migrate. In adherent, non-muscle cells such as endothelial cells and fibroblasts, tension is a result of actomyosin stress fibers generating forces that are resisted by cell-matrix adhesions. Stretching the matrix upon which cells adhere perturbs the cell-matrix traction forces and cells respond by actively re-establishing the pre-existing level of force [1], [2]. Fiber tension extends stress fibers beyond their unloaded lengths and cells maintain fiber strain at an optimal level that depends on actomyosin activity [3]. Sudden large (>20%) increases or decreases in matrix strain result in rapid stress fiber disassembly and reassembly [3], [4], [5], suggesting that perturbing fiber strain from the optimal level increases the rate of stress fiber turnover.

When cyclically stretched at frequencies at or above 1 Hz, cells and their stress fibers tend to orient away from the direction of stretch, but remain randomly oriented when subjected to stretch at low frequencies [6], [7]. Theoretical analyses indicate that the frequency dependence of stretch-induced stress fiber alignment is a result of the competition between the rate of change in fiber tension due to the applied strain and the rate of active fiber relaxation caused by myosin sliding [7], [8]. At low stretch frequencies, perturbations in tension are predicted to relax quickly so that fiber tension remains constant despite cyclic changes in fiber length. At high stretch frequencies, the stress fibers cannot relax quickly enough to dampen the changes in fiber tension; hence, the stress fibers are expected to undergo a rapid increase in turnover. Over time, the levels of stress fiber turnover and cytoskeletal tension are predicted to decrease as stress fibers accumulate in the direction generating the lowest stress or strain.

Cyclic stretching of endothelial cells (ECs), such as occurs in arteries, activates several proteins involved in the regulation of gene expression, including the mitogen-activated protein kinases (MAPKs) [9]. Members of the MAPK family include c-Jun NH_2_-terminal kinase (JNK), extracellular signal-regulated kinase (ERK) and p38. JNK and p38 kinases are thought to be among the major regulators of pro-atherogenic inflammatory gene expression in ECs, while ERKs are primarily involved in cell growth and survival [10]. In arteries, ECs and their stress fibers are oriented perpendicular to the principal direction of cyclic circumferential stretching and parallel to the direction of blood flow [11]. The lack of EC alignment at arterial branch points and curvatures is associated with atherogenesis, suggesting that cell alignment is somehow important in maintaining an anti-atherogenic cell phenotype [12]. JNK, ERK and p38 are activated by cyclic stretch in bovine pulmonary ECs, and inhibition of any of these MAPKs attenuates activation of the AP-1 transcription element, but does not affect stretch-induced cell alignment [9]. Although JNK does not appear to regulate stretch-induced cell alignment, stress fiber alignment perpendicular to the direction of cyclic stretch results in suppression of stretch-induced JNK activation in bovine aortic ECs [13]. We have reported that a low dose of cytochalasin D greatly diminishes the number and size of stress fibers in ECs and the basal level of JNK activity [13], which may be due to decreased cytoskeletal tension. JNK and ERK activations are quantitatively related to tension in rat skeletal muscle preparations subjected to stretch [14]. Together, these studies suggest that the time course of cyclic stretch-induced MAPK activations may be regulated by temporal changes in cytoskeletal tension as stress fibers align perpendicular to the direction of stretch.

Focal adhesions are interesting protein complexes since they serve to both transduce mechanical signals and mechanically link the actin cytoskeleton to the extracellular matrix. Integrins contained within focal adhesions are activated into a high-affinity conformation by extracellular binding and tension [15]. Phosphorylation of focal adhesion kinase (FAK) in response to integrin activation initiates signaling through multiple intracellular pathways that regulate cell motility, gene expression and cell proliferation [16]. Wang and colleagues [17] provided evidence that FAK is a force sensor since FAK-expressing mouse embryonic fibroblasts (MEFs) redirect their migration in response to local changes in matrix tension, while FAK-null MEFs do not. The level of FAK phosphorylation transiently increases in response to cyclic stretch in ECs [18] and fibroblasts [19]. FAK is reportedly required for stretch-induced phosphorylation of JNK [20], ERK and p38 [21], [22], as well as stretch-induced cell alignment [18]. However, FAK is not always required for EC mechanotransduction [23]. In the present study, we demonstrate that stretch-induced JNK, p38 and ERK phosphorylation and stress fiber reorganization depend on the rate of applied matrix strain. Further, our results provide evidence that stretch-induced JNK, p38 and ERK phosphorylation and actin reorganization are driven by perturbations in cytoskeletal tension and are not mediated by FAK.

## Materials and Methods

### Constrained Mixture Model of Stress Fiber Networks

Stretch-induced changes in stretch in stress fibers were estimated using a stochastic constrained mixture model of stress fiber networks described previously [7]. The C++ computer code used to solve the model can be found in Text S1. This model predicts the effects of cyclic matrix stretching and stress fiber remodeling on stress fiber strain. Under static conditions, stress fibers in endothelial cells are pre-extended due to actomyosin contractility such that the ratio of the fiber length under tension and the unloaded length is 1.10 [3]. Thus, the simulations were performed starting with an ensemble of *N* randomly oriented stress fiber all with a stretch ratio *α*_0_ = 1.10. The level of stretch for each fiber changes in response to matrix stretching. The deformation gradient tensor **F**(t, Δ*t*) describes the deformation of the matrix from an initial configuration at time *t* to a new configuration at time *t*+Δ*t*. The stretch ratio of a line element on the matrix in the direction of the *i*th fiber is
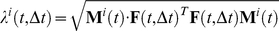
(1)where **M** *^i^* is the unit vector in the direction of the *i*th fiber at time *t* [24]. Motivated by experimental evidence of stress fiber viscoelastic properties [25], we assumed stress fibers relax at a rate proportional to the perturbation in fiber stretch,
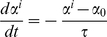
(2)where *τ* is the relaxation time constant [7]. For incremental step changes in strain, the stretch ratio of fiber *i* can be integrated numerically as
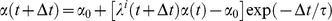
(3)To maintain numerical stability, the time intervals were kept small relative to the rate of relaxation (i.e. Δ*t*≤0.01τ).

The dynamic turnover of stress fibers was described using a stochastic approach with the probability of a fiber disassembling over a time interval Δ*t* expressed as
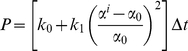
(4)The disassembly of a fiber is immediately followed by the assembly of a new stress fiber with a stretch 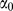 oriented in a randomly chosen direction. The initial value problems were solved numerically for cyclic and step changes in stretch using model parameters (*k*_0_ = 3.0×10^−4^ sec^−1^, *k*_1_ = 1.8×10^3^ sec^−1^, and *τ* = 0.5 sec) previously determined by fitting measured time courses of stress fiber alignment in non-confluent BAECs [7].

### Cell Culture

Bovine aortic endothelial cells (BAECs; Lonza), U2OS osteosarcoma cells (MarinPharm GmbH), and FAK-null (FAK −/−) and FAK-expressing (FAK +/+) mouse embryonic fibroblasts (MEFs) were cultured in DMEM (GIBCO) supplemented with 10% fetal bovine serum, 2mM L-glutamine, 1mM sodium pyruvate and 1mM penicillin/streptomycin as described previously [13], [26]. Stretch chambers were sterilized with UV radiation and coated with 1µg/cm^2^ fibronectin (Sigma) overnight. The cells were seeded onto the membranes, allowed to spread in complete media, and serum-starved overnight prior to stretching.

### Stretch Experiments

For immunoblotting and immunostaining experiments, BAECs and MEFs were stretched using a device previously described [7], [13]. Briefly, silicone rubber membranes were attached to polycarbonate chambers with O-rings. The device is capable of generating cyclic stretch in a frequency range of 0.01 to 1 Hz and was set to produce a 10% linear stretch. Depending on the geometry of the Teflon indenter used, the device produces either pure uniaxial stretch (stretch in the principal direction without stretching in the orthogonal direction) or equibiaxial stretch (stretch is equal in all directions in the 2-dimensional plane of stretching). Shear stresses due to the movement of fluid in the stretch chamber are estimated to be <0.2 dynes/cm^2^, hence are not expected to affect the signaling pathways investigated in this study [13]. The device was kept in a humidified 5% CO_2_-95% air incubator at 37°C.

For live microscopy experiments, U2OS cells stably transfected with GFP-actin (MarinPharm, Germany) were stretched on silicone rubber chambers (STREX, Japan) using Hyclone L-15 CO_2_-independent media (Fisher Scientific). The equibiaxial chambers (STREX, ST-CH-04-XY) were stretched using four linear motors (Parker Motions). The entire stretch apparatus was mounted on the stainless steel stage (Gibraltar) of a Nikon FN1 upright microscope and imaged with a Nikon C1 laser scanning confocal head with a 60× water-dipping objective illuminated with a 40-mW Argon ion laser (Melles Griot). The stretch device and microscope were housed in a custom-made acrylic enclosure maintained at 37°C using a heat gun (Omega) regulated by a temperature controller (Omega). For cyclic stretching experiments, the stretch cycle was stopped periodically for approximately 30 seconds to focus and capture an image while the chamber was in the unstretched configuration.

### Immunoblotting

Cells were lysed in 2× Laemmli buffer (Sigma). Samples were run on a 10% SDS-PAGE gel, transferred onto PVDF membranes (Bio-Rad) and blocked with 5% milk in TBS with 0.01% Tween-20. Blots were incubated (1∶1000) with phosphorylation site-specific primary antibodies directed against T183/Y185 JNK, T202/Y204 ERK and T180/Y182 p38 (Cell Signaling) at 4°C overnight. Blots were washed, incubated with 1∶3000 secondary goat anti-rabbit antibody (Jackson Laboratories) for 30 min at room temperature and developed using ECL reagents (Pierce) and film (Denville Scientific). Immunoblots used to measure phospho-specific MAPK protein were stripped and reprobed with the respective primary pan antibody directed against JNK, ERK and p38 (Santa Cruz) to normalize the measured levels of phosphorylated MAPK relative to the respective measured levels of total MAPK protein in each sample. The values were then normalized by the values for the respective static controls.

### Quantification of Stress Fiber Organization

Cells were subjected to stretch, rinsed with PBS at 37°C, fixed in 4% paraformaldehyde in PBS for 10 min at room temperature, and permeabilized with 0.5% Triton X-100 in PBS for 15 min. Actin filaments were then labeled with Alexa 488-phalloidin (Invitrogen) for 45 min at 1∶200 dilution in PBS. Images were captured by confocal microscopy as described above. The images were post-processed using a custom-made algorithm in MATLAB (the MathWorks, Natick, MA) to determine, for each image or cell, a density distribution 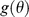 of the stress fiber orientations [7], [13]. An order parameter
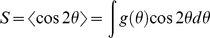
(5)was calculated for each image (for confluent BAEC monolayers) or each individual cell (for non-confluent MEFs) to characterize the dispersion in SF orientations. Values for *S* of 0, 1 or −1 indicate the stress fibers are uniformly distributed in all directions, uniformly oriented parallel to the stretch direction, or uniformly oriented perpendicular to the stretch direction, respectively.

### Quantification of Cell Spreading Area

GFP-actin expression in U2OS cells localizes to stress fibers, but also results in some diffuse fluorescence throughout the cytoplasm. Cell outlines were determined from images of GFP fluorescence in the U2OS cells by image thresholding at a grayscale level 5 units greater than the noise in the background surrounding the cell. Cell areas were then calculated using the Analyze Particles macro in ImageJ (NIH) with the values then normalized relative to the original area of the cell in an image captured immediately before initiating cyclic stretch.

### Statistical analyses

Statistical differences in the levels of phosphorylation, the order parameters (cf., Eqn. 5), or cell area between groups were evaluated by ANOVA followed by Student-Newman-Keuls posthoc multiple comparison testing.

## Results and Discussion

### Cyclic uniaxial stretch induces stress fiber alignment in a strain rate-dependent manner

**Figure 1:**
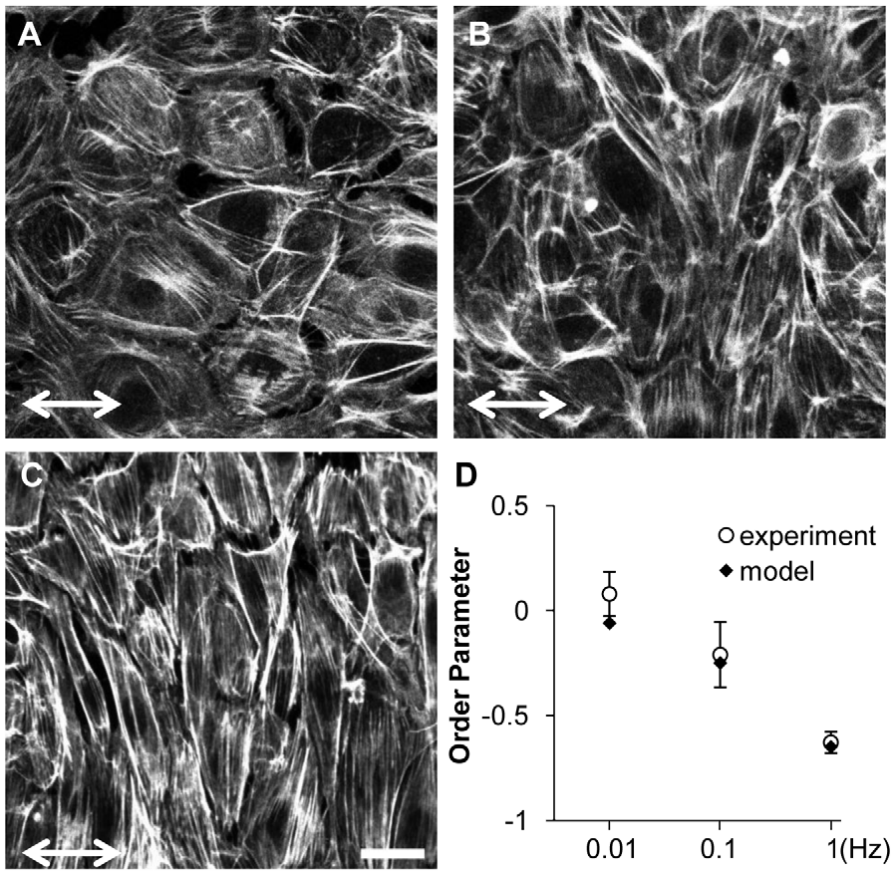
Cyclic stretch-induced stress fiber alignment depends on stretch frequency. **A–C**: Representative images of confluent BAECs subjected to 4 hours of 10% cyclic uniaxial stretch at frequencies of 0.01 (**A**), 0.1 (**B**) and 1 Hz (**C**) in the direction indicated. After the experiments, cells were fixed and then stained with Alexa 488-phalloidin to identify stress fibers. **D**: Order parameters were computed for each image to quantify the extent of stress fiber alignment and the results were summarized (mean ± S.D.; n = 25). Order parameters were also computed for simulated stretch-induced stress fiber reorganization under the same stretch conditions for comparison to the experimental results. Scale bar length is 20 µm.

Our theoretical model of stretch-induced stress fiber reorganization predicts that stress fibers gradually orient perpendicular to the direction of cyclic uniaxial stretch (10% stretch, 1Hz) to minimize the amplitude of fiber strain [7]. We subjected confluent BAECs to 4 hours of 10% cyclic uniaxial stretch at different strain rates by stretching at frequencies ranging from 1 to 0.01 Hz. We then fixed the cells, stained F-actin with Alexa 488-phalloidin, and quantified the extent of stress fiber alignment. Consistent with our previous results using non-confluent BAECs [7], there was no alignment at 0.01Hz (Fig. 1A) and the extent of alignment perpendicular to the direction of matrix stretch increased as frequency increased to 0.1 and 1 Hz (Figs. 1 B and C). Figure 1D illustrates that there was a significant decrease in the order parameter for each frequency (p<0.05, alignment at 1Hz>0.1Hz>0.01Hz). Thus, the extent of stress fiber reorganization decreased as the rate of matrix strain decreased.

**Figure 2:**
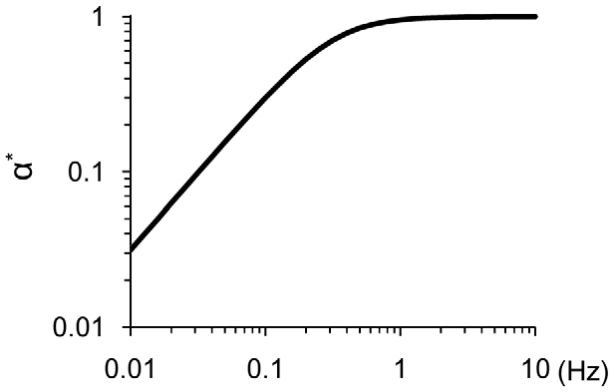
Stress fiber stretch amplitude depends on stretch frequency. The relative fiber stretch amplitude α^*^ was estimated by solving the model for the initial cycle of 10% cyclic uniaxial or equibiaxial stretch for frequencies ranging from 0.01 to 10 Hz. The simulations predict that the stress fibers behave elastically above a saturation frequency of ∼1Hz and that the value of α^*^ decreases exponentially as frequency decreases below the saturation frequency.

Changes in cytoskeletal tension caused by cyclic stretch have not been measured directly; however, we can estimate changes in stress fiber stretch as a correlate of tension using our mathematical model. Simulations of stretch-induced stress fiber reorganization under these same cyclic uniaxial stretch conditions resulted in order parameter values very similar to those calculated from the experimental data (Fig. 1D).To quantify the effects of fiber relaxation on fiber stretch, we define the relative fiber stretch amplitude α* as the amplitude of fiber stretch during a stretch cycle divided by the amplitude of matrix stretch in the same direction. The value of α* ranges from 0 for a fully relaxed fiber to 1 for a fiber that has not relaxed. We computed α* as a function of stretch frequency during the first cycle of stretch, i.e. before stress fiber alignment contributes to decreasing fiber stretch (Fig. 2). At stretch frequencies greater than 1 Hz, the rate of change in fiber length is estimated to be much faster than the rate of relaxation, thus α* approaches unity. As stretch frequency decreases, fiber relaxation becomes increasingly significant. Consequently, the amplitude of fiber tension decreased by 60% at 0.1 Hz and by 97% at 0.01 Hz. Thus, decreasing the frequency of stretching (i.e. decreasing the rate of fiber strain) decreased the extent of fiber alignment, which we attribute to fiber relaxation.

### Cyclic equibiaxial stretch decreases cell spreading in a strain rate-dependent manner

**Figure 3:**
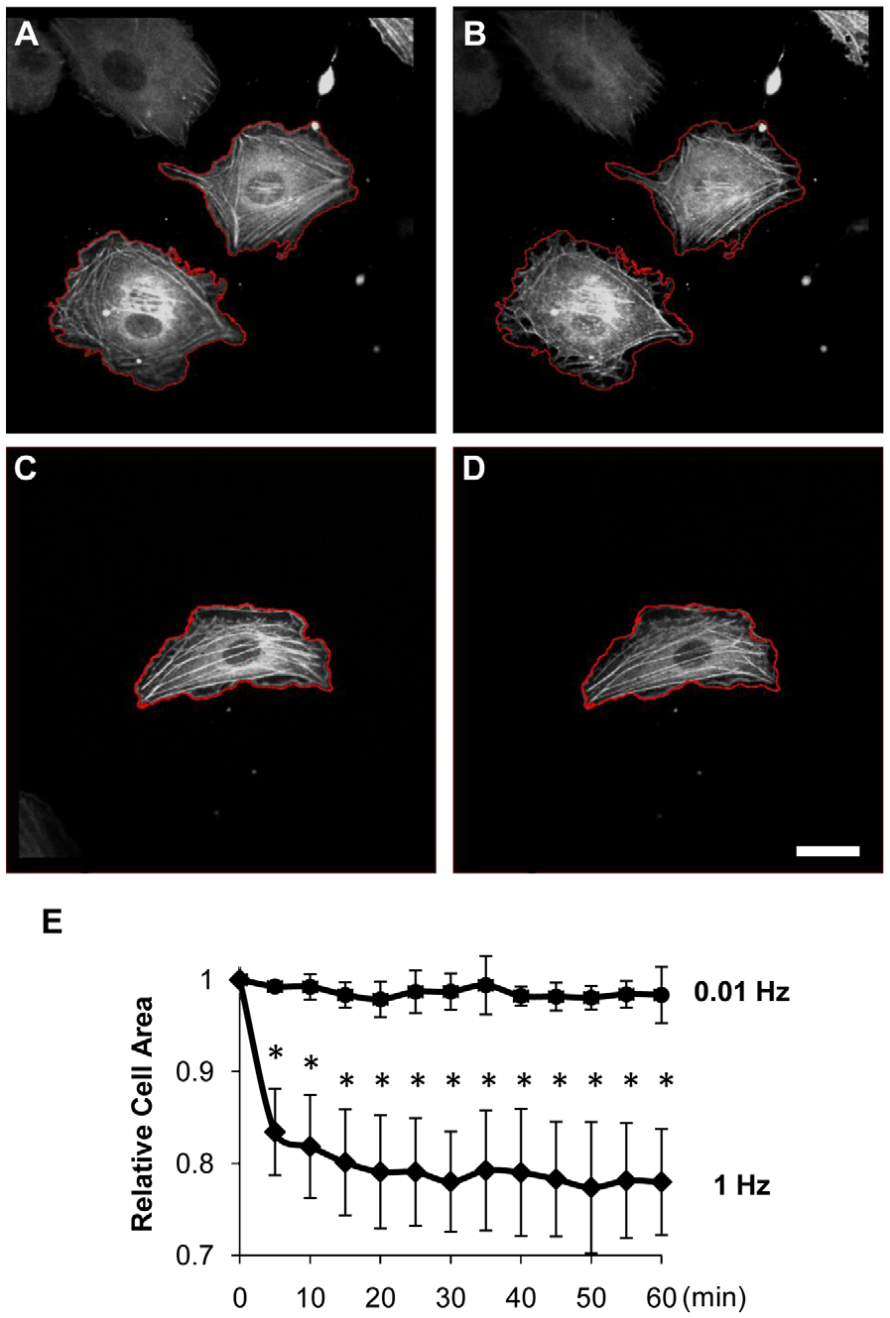
Cell retraction caused by 1Hz, but not 0.01Hz, cyclic equibiaxial stretch. Representative images are shown of U2OS cells expressing GFP-actin imaged immediately before (**A**, **C**) and 5 min after applying 10% equibiaxial stretch at 1Hz (**B**) or 0.01 Hz (**D**). The outlines of the cells prior to stretching (red) are shown for reference. Bar = 20 µm. **E**: The ratio of cell areas (after/before stretch) for the indicated durations of stretch at 1Hz and 0.01Hz are summarized (mean±S.D.; n = 6). * indicates significant difference from unity (*P*<0.01).

Cyclic equibiaxial stretch at 1Hz does not induced stress fiber alignment [13]. However, there is no report on the dynamic response of stress fibers in cells subjected to cyclic equibiaxial stretch. We collected time-lapse images of U2OS cells expressing GFP-actin subjected to 10% stretch at 1 and 0.01 Hz. The initial image for each condition was collected and cyclic stretch was then immediately initiated (Fig. 3A). Thereafter, images were collected every five minutes with the images taken in the reference configuration of the substrate (i.e. the unstretched position). Unlike the rapid disassembly and reassembly of stress fibers within minutes caused by cyclic uniaxial stretch reported by Hayakawa and colleagues [27], there was no obvious increase in the rate of stress fiber disassembly and reassembly when the cells were subjected to cyclic equibiaxial stretch at 1Hz (Video S1). The cells did, however, consistently retract to ∼80% of their original area after 5 minutes (Fig. 3B) and maintained this retracted area throughout the 60 minute experiment (Fig. 3E). Our model predicts that cyclic equibiaxial stretch at 1Hz generates a large increase in the amplitude of fiber stretch (cf. Fig. 2), which may induce the sustained cell retraction. Consistent with this hypothesis, cyclic equibiaxial stretching at 0.01Hz, which is predicted to generate a negligible perturbation in fiber tension (cf. Fig. 2), did not cause a significant change in cell spreading area (Fig. 3C–E and Video S2).

### Cyclic stretch increases MAPK phosphorylation levels in a strain rate-dependent manner

**Figure 4:**
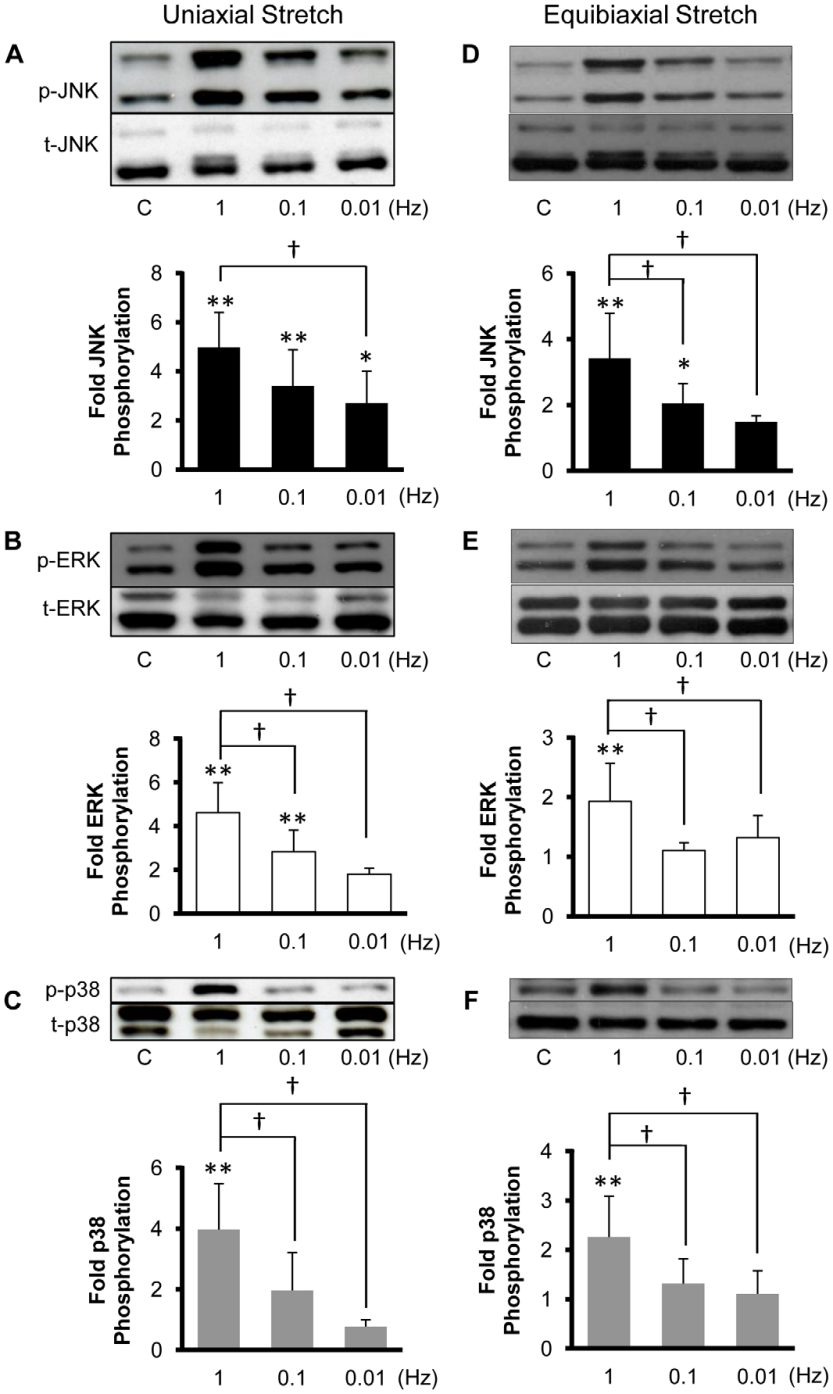
JNK, ERK and p38 phosphorylation depend on the frequency of cyclic stretch. Representative Western blots of phospho-specific and total JNK (**A**, **D**), ERK (**B**, **E**) and p38 (**C**, **F**) were obtained from confluent BAECs kept as static controls or subjected to 10% cyclic uniaxial (**A–C**) or equibiaxial (**D–F**) stretch for 30 min at the indicated frequencies. Optical density measurements were quantified to determine relative amounts of phosphorylated MAPK normalized by the respective total MAPK. The values (means±S.D.; n = 6 for uniaxial and n = 7 for equibiaxial) indicate the fold change in phosphorylation relative to the static control for each individual experiment. * and ** indicate significant difference from static controls (* *P*<0.05, ** *P*<0.01). † indicates significant difference between groups stretched at different frequencies (*P*<0.05).

Qu et al. [28] subjected vascular smooth muscle cells to 10% cyclic uniaxial stretch at frequencies of 0, 0.5, 1 and 2 Hz and reported a peak in ERK phosphorylation at 0.5 Hz and a peak in p38 phosphorylation at 1 Hz. Over this same range of stretch frequencies, Hosokawa et al. [29] reported a monotonic increase in ERK activation with near maximal activation at 1Hz in vascular smooth muscle cells subjected to 20% cyclic uniaxial stretch. We previously demonstrated that JNK is activated in confluent BAECs subjected to 30 min of 10% cyclic uniaxial and equibiaxial stretch at a frequency of 1 Hz [13]. We hypothesized that the levels of phosphorylation of the MAPKs show a similar dependence on stretch frequency as stretch-induced stress fiber alignment (cf. Fig. 1) and cell retraction (cf. Fig. 3). To test this hypothesis, BAECs were subjected to 30 minutes of 10% cyclic uniaxial (Fig. 4A–C) and equibiaxial stretch (Fig. 4D–F) at frequencies of 0.01, 0.1 and 1 Hz and the levels of JNK, ERK and p38 phosphorylation were quantified. Stretching at 1 Hz resulted in significant increases in the activities of JNK, ERK and p38 relative to static controls. Further, the extent of activation of each MAPK decreased significantly as the frequency of stretch was decreased to 0.01 Hz. Thus, JNK, ERK and p38 phosphorylation levels increase monotonically with strain rate (i.e. stretch frequency) in BAECs subjected to either uniaxial or equibiaxial stretch.

### Transient changes in fiber strain rate induce transient changes in JNK and ERK phosphorylation levels, but do not induce stress fiber reorganization

**Figure 5:**
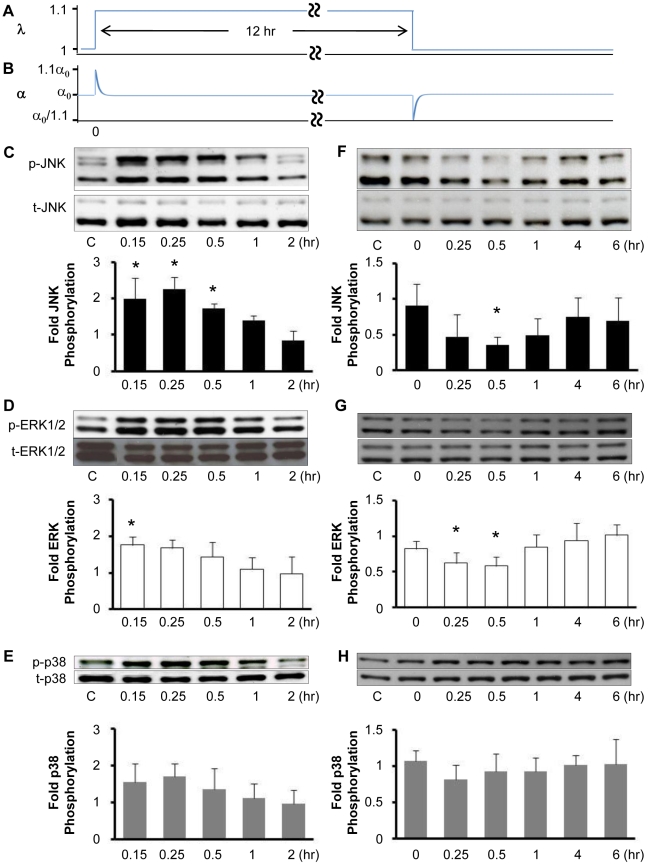
Transient changes in strain rate induce transient changes in the levels of JNK, ERK and p38 phosphorylation. Confluent BAECs were subjected to a stretch release maneuver consisting of 10% equibiaxial stretch *λ*, followed by the release of the stretch 12 hr later (**A**), which was predicted to generate a transient increase and subsequent transient decrease in fiber stretch *α* (**B**). Representative Western blots of phospho-specific and total JNK (**C**, **F**), ERK (**D**, **G**) and p38 (**E**, **H**) from cell lysates collected at the indicated times after the initial stretch (**C**–**E**) and subsequent stretch release (**F**–**H**). Optical density measurements were quantified to determine relative amounts of phosphorylated MAPK normalized by the respective total MAPK. The values (means±S.D.; n = 4) indicate the fold change in phosphorylation relative to static controls for each individual experiment. * indicates significant difference from static control (*P*<0.05).

Gavara and colleagues [1] reported that cell-matrix traction forces increase in response to a step increase in equibiaxial stretch, but later, after the stretch is released, traction forces drop below baseline levels and gradually return toward baseline levels over a period of ten minutes. These authors attributed the changes in traction forces to changes in actin cytoskeletal tension. We applied our model to interpret these results and to predict the effects of a step equibiaxial stretch followed by a release on fiber stretch. Solving Eqn. 3 for the case of a single step change in equibiaxial stretch of magnitude *λ* demonstrates that the model predicts an initial elastic change in fiber stretch (*λα*_0_−*α*_0_) that relaxes back to the original stretch *α*_0_ at a rate dependent on *τ*:
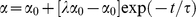
(6)Note that the indices *i* are neglected here since the stretching is isotropic and all stress fibers are predicted to behave identically for equibiaxial stretch. As shown in Figure 5B, a 10% step increase in equibiaxial stretch (*λ* = 1.1) is predicted to result in an elastic increase in fiber stretch from *α*_0_ to 1.1*α*_0_ that quickly returns to *α*_0_. Later, releasing matrix stretch then causes an elastic decrease in fiber stretch to *α*_0_/1.1 that also quickly returns to *α*_0_. Assuming that fiber stretch is proportional to average traction force, these predictions are consistent with the changes in traction force caused by stretch and release measured by Gavara and colleagues [1], with a time constant on the order of several minutes. This is a much larger value for the time constant than the value of 0.5 sec found to describe the frequency dependence of cyclic stretch-induced stress fiber alignment (cf. Fig. 1D). For the case of a step change in stretch, a value for *τ* determined from experimental measurements is likely to be more accurate than the one extracted from the model parameter fitting to cyclic stretch data. Regardless of the discrepancy in the value for *τ*, the model predicts full stress fiber relaxation within minutes.

Motivated by our predictions of stress fiber relaxation, we hypothesized that a step increase in equibiaxial stretch would cause a transient increase in MAPK phosphorylation levels. Further, we hypothesized that releasing this stretch would cause a transient decrease in MAPK phosphorylation levels. First, we subjected BAECs to a 10% step equibiaxial stretch and measured changes in the levels of phosphorylated JNK, ERK and p38 (Fig. 5C–E). The levels of phosphorylation of JNK and ERK significantly increased at 0.15 hours and then subsided after 1 to 2 hours. A small increase in the average levels of p38 phosphorylation was also observed, however the increase was not statistically significant. These results are consistent with the transient increases in the activities of JNK, ERK, but not p38, reported for cardiac fibroblasts subjected to a 4% step increase in equibiaxial stretch [30]. Next, we subjected BAECs to a 10% step equibiaxial stretch, 12-hour hold, and stretch release maneuver (c.f., Fig. 5A) and quantified the levels of phosphorylation of the MAPKs over time. The release in stretch resulted in a transient decrease in the levels of JNK and ERK phosphorylation, reaching a minimum at 0.5 hours, and returning to near baseline levels after 4 hours (Fig. 5F and G). The step stretch-release maneuver did not result in a measureable change in p38 activity, however (Fig. 5H). It is not clear why the level of JNK phosphorylation did not fully return to the baseline level, however the difference is not statistically significant. Thus, a transient increase in the rate of strain caused a transient increase in JNK and ERK phosphorylation, while a transient decrease in the rate of strain caused a transient decrease in JNK and ERK phosphorylations.

Step equibiaxial stretch-induced JNK activation in fibroblasts has been reported to require the formation of new integrin-matrix bonds [15]. Since stress fiber assembly requires the formation of new integrin-matrix bonds, we tested if stretch-induced JNK and ERK activations by stretch may be related with stress fiber reorganization. We collected time-lapse videos of U2OS cells expressing GFP-actin subjected to a single 10% step equibiaxial stretch (Video S3) and to a 10% step equibiaxial stretch and release 4 hours later (Video S4).Some previous reports indicate that transient stretches induce sustained fluidization of the cytoskeleton [5], [31]. In our experiments, stress fibers did not undergo significant disassembly and reassembly in response to either a step equibiaxial stretch or an equibiaxial stretch release maneuver. These results are consistent with the report that the content of filamentous actin in osteoblastic cells is not changed by either uniaxial 10% step stretch or stretch release [32]. It is possible that larger stretch magnitudes would result in greater changes to the cytoskeleton. Given that Gavara et al. [1] measured an increase in cell-matrix traction force in response to equibiaxial step stretch and a transient decrease in cell-matrix traction forces in response to equibiaxial step stretch-release, these results suggest that step stretch-induced changes in MAPK activities correlate with changes in traction forces, but not stress fiber turnover. We cannot rule out a role for integrin-matrix bond turnover, however. Since stress fibers transmit traction forces to the extracellular matrix, these results do imply that JNK and ERK phosphorylation levels correlate with changes in stress fiber tension. Traction forces can reportedly induce integrin bond turnover without appreciable remodeling of stress fibers [33].

### Stress fiber inhibitors decreases JNK and ERK phosphorylation levels

**Figure 6:**
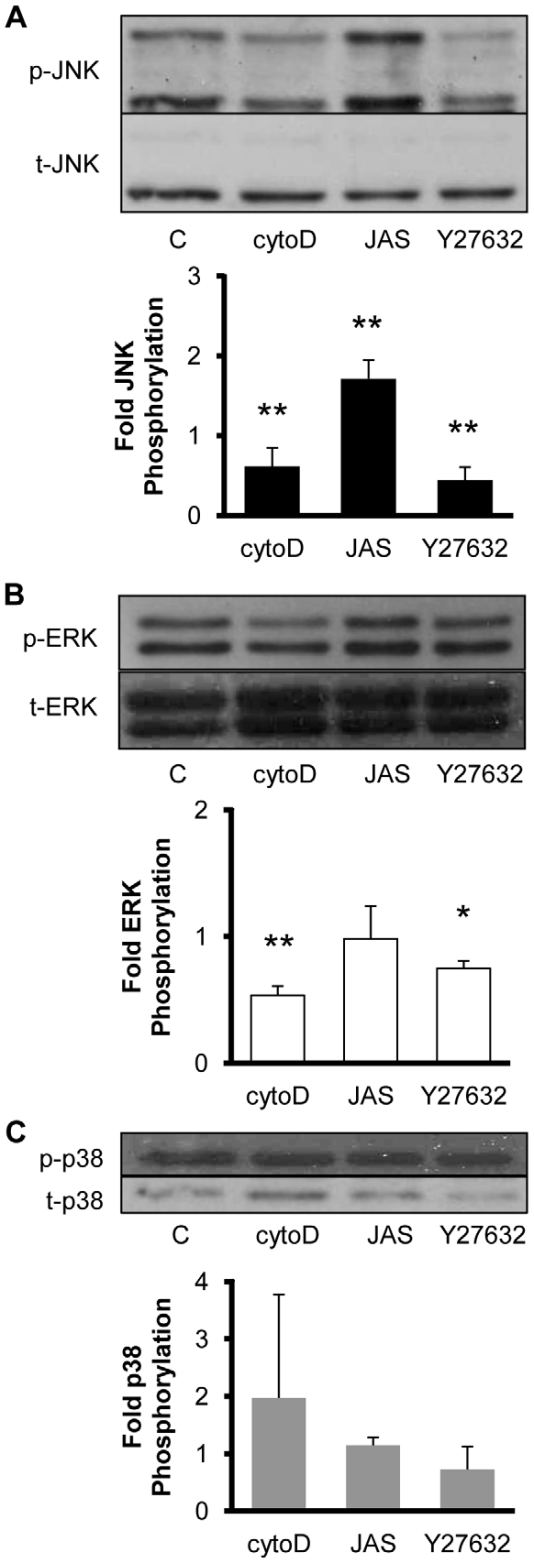
JNK and ERK phosphorylation is decreased by inhibitors of Rho kinase and actin polymerization. Representative Western blots are shown of phospho-specific and total JNK (**A**), ERK (**B**) and p38 (**C**) from cell lysates collected from confluent BAECs treated for 30 min with vehicle (0.1% DMSO; lane 1), 50 nM cytochalasin D, 10 nM jasplakinolide or 10 µM Y27632. Optical density measurements were quantified to determine relative amounts of phosphorylated MAPK normalized by the respective total MAPK. The values (means±S.D.; n = 4) indicate the fold change in phosphorylation relative to the DMSO-treated controls for each individual experiment. * and ** indicate significant difference from the control (* *P*<0.05, ** *P*<0.01).

Stress fiber contractile function requires the bundling of actin filaments and actomyosin contractile activity. To further explore the role of stress fiber tension on MAPK phosphorylation, we treated BAECs with cytochalasin D (50nM) to disrupt actin filaments and Y27632 (10µM) to reduce actomyosin activity. Cytochalasin D and Y27632 significantly reduced the phosphorylation levels of JNK and ERK, but not p38, compared to the levels in BAECs treated with the DMSO vehicle alone (Fig. 6). BAECs were also treated with jasplakinolide (10nM) to stabilize actin filaments, which is expected to maintain stress fiber integrity in bovine ECs without significantly changing basal cell contractile tone [34]. Jasplakinolide treatment caused an increase in the level of JNK phosphorylation without significantly affecting p38 and ERK. These results are consistent with JNK and ERK phosphorylation levels being regulated by cytoskeletal tension since inhibitors than disrupt stress fiber stability or contractility decrease JNK and ERK phosphorylation levels. Further, these results provide additional evidence that MAPK activities are not associated with stress fiber turnover since stabilizing filamentous actin does not reduce MAPK phosphorylation levels.

### FAK is not required for stretch-induced changes in stress fiber alignment in MEFs

FAK is thought to be a force sensor that undergoes force-induced conformational changes that can regulate protein activity [35]. FAK is reportedly necessary for normal focal adhesion turnover and mechanosensing [17], [36]. Relative to their FAK-expressing counterparts, FAK-null keratinocytes are less motile and have less dynamic stress fibers and focal adhesions [37]. These studies strongly indicate that FAK is a major protein regulating stretch-induced stress fiber reorganization. On the other hand, our model of stretch-induced stress fiber reorganization is based on the argument that the stress fibers reorient in direct response to perturbations in cytoskeletal tension. This premise is supported by the observation that large step increases in stretch can cause fragmentation and subsequent disassembly of stress fibers [3]. Thus, it is unclear if FAK mediates changes in stress fiber organization in response to cyclic stretch.

**Figure 7:**
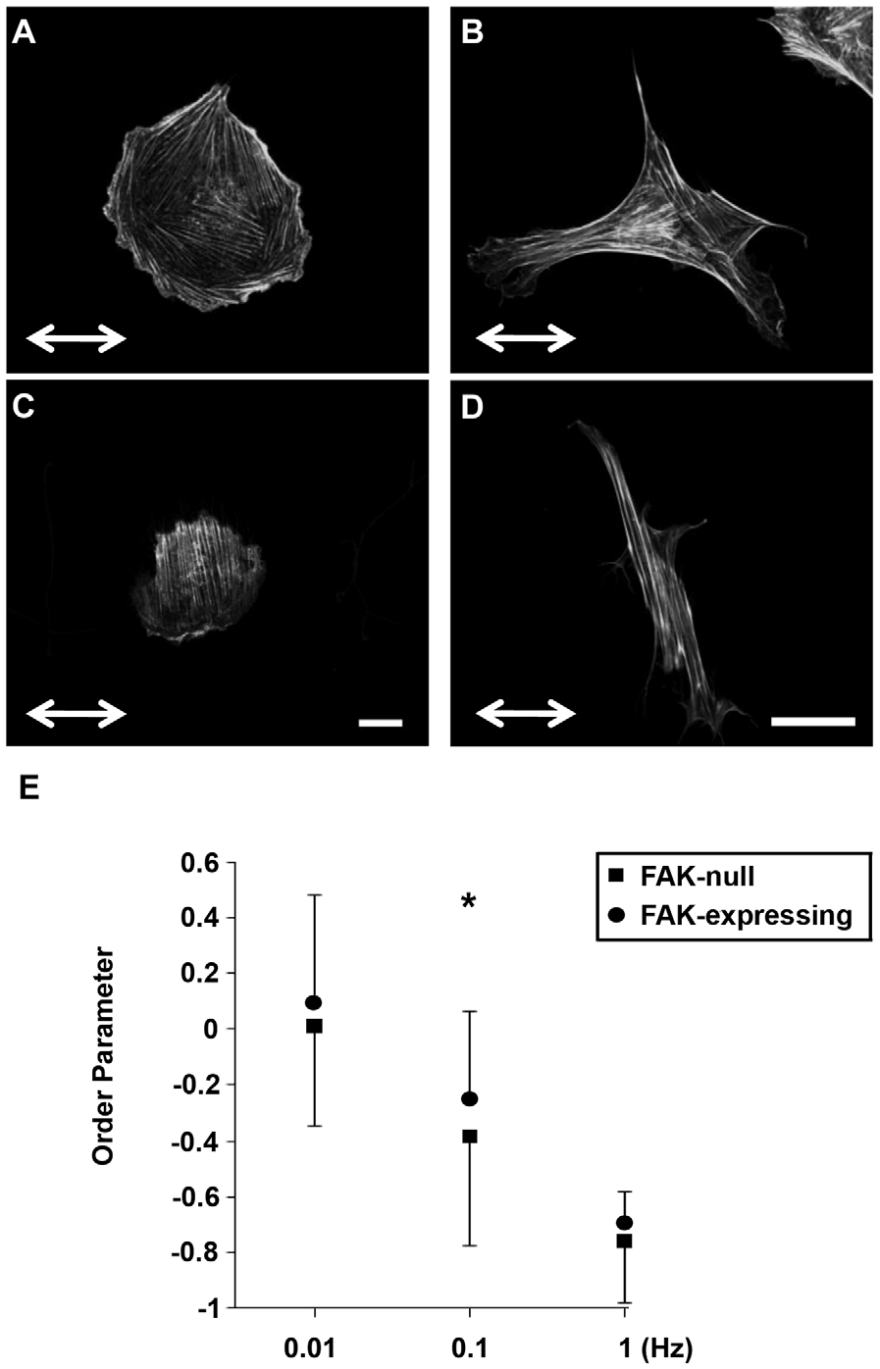
FAK is not necessary for stretch-induced stress fiber alignment. Representative micrographs are shown from experiments in which non-confluent FAK-null (**A**, **C**) and FAK-expressing (**B**, **D**) MEFs were subjected to 3 hours of 10% cyclic uniaxial stretch at 0.01 (**A**, **B**) or 1 Hz (**C**, **D**) in the direction indicated, fixed and stained to measure stress fiber reorganization. **E**: Experiments were performed at frequencies of 0.01, 0.1 and 1 Hz and the order parameters for individual cells from three different experiments were quantified (mean±SD; n = 75). Bar = 20 µm. * indicates a significant difference between frequency-matched groups, (*P*<0.05).

We evaluated the role of FAK in regulating stretch-induced stress fiber alignment using fibroblasts obtained from normal and FAK knockout mouse embryos. FAK-null and FAK-expressing MEFs were subjected to 3 hours of 10% cyclic uniaxial stretch at 0.01, 0.1 or 1 Hz, fixed, stained with Alexa 488-phallodin, and imaged by confocal microscopy. FAK-null MEFs subjected to cyclic stretch at 0.01 Hz (Fig. 7A) generally contained fewer protrusions and were less polarized than FAK-expressing MEFs stretched under the same conditions (Fig. 7B). Following cyclic stretching at 1 Hz, stress fibers strongly aligned perpendicular to the direction of cyclic stretch in both FAK-null (Fig. 7C) and FAK-expressing MEFs (Fig. 7D). However, the FAK-null cells subjected to stretch at 1 Hz appeared to be generally less elongated than the FAK-expressing cells. Such diminished cell elongation may have contributed to the conclusion by Naruse and colleagues [18] that suppression of FAK expression using antisense RNA inhibits cyclic stretch-induced alignment of human umbilical vein ECs. Order parameters were calculated (Fig. 7E) and multi-comparison testing indicated that 1) there were significant differences (p<0.05) in order parameter for each frequency for both cell types (alignment at 1Hz>0.1Hz>0.01Hz); and 2) stress fiber alignment was significantly higher in FAK-null MEFs at the sub-threshold frequency of 0.1 Hz, although the difference was not remarkable. These results support the hypothesis that cyclic stretch-induced stress fiber reorganization occurs through destabilization of stress fiber integrity rather than through force sensing by FAK. Namely, the failure of a stress fiber may unload the associated focal adhesions, leading to their disassembly. These focal adhesions would later be reassembled during the formation of a new stress fiber.

### FAK is not required for stretch-induced changes in MAPK phosphorylation in MEFs

**Figure 8:**
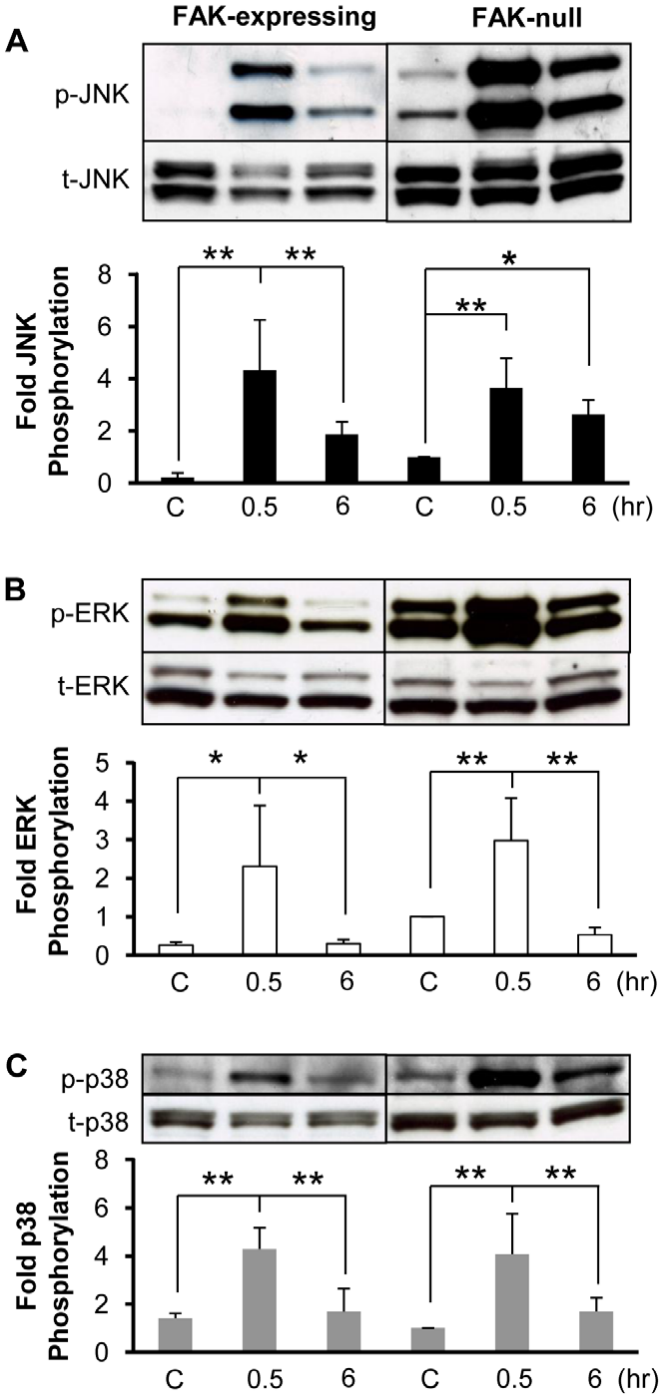
FAK is not necessary for stretch-induced increases in JNK, ERK and p38 phosphorylation. Representative immunoblots are shown from experiments in which confluent FAK-expressing and FAK-null MEFs were kept as static controls or subjected to 10% cyclic uniaxial stretch at 1 Hz for 0.5 and 6 hr. Immunoblot pairings for JNK (**A**), ERK (**B**) and p38 (**C**) for the FAK-expressing and FAK-null MEFs were taken from two locations on the same blot. Optical density measurements were quantified to determine relative amounts of phosphorylated MAPK normalized by the respective total MAPK. The values (means±S.D.; n = 4) indicate the fold change in phosphorylation relative to the FAK-null static control for each individual experiment. * and ** indicates significant difference between groups (* *P*<0.05, ** *P*<0.01).

There is also substantial evidence indicating that FAK is required for stretch-induced MAPK activations [20], [21], [22]. We subjected FAK-null and FAK-expressing MEFs to 10% cyclic uniaxial stretch at 1 Hz and measured the levels of phosphorylation of the MAPKs. The levels of phosphorylated JNK, ERK and p38 were each transiently increased after 30 min of cyclic stretch in both FAK-null and FAK-expressing MEFs (Fig. 8). While the absolute levels of phosphorylated MAPKs was higher in the FAK-null MEFs (Fig. 8, upper blots), this was attributed to higher levels of total JNK, ERK and p38 expression (Fig. 8, lower blots). Thus, FAK is not necessary for stretch-induced activation of JNK, p38 and ERK.

### Summary

It is becoming increasingly clear that cytoskeletal tension plays a major role in maintaining normal cell function, and that loss of ‘tensional homeostasis’ promotes disease progression, including fibrosis, cancer and atherosclerosis [38]. While stretching stress fibers increase their tension [39], stress fibers display viscoelastic properties [25] that allow relaxation of perturbations in tension. Dynamic changes in cytoskeletal structure, mechanical properties, and intracellular signaling all depend on the spatio-temporal pattern of externally applied forces as well as active responses within the cell. Theoretical modeling provides a valuable tool to make sense of this complex system. The present study, which was designed and interpreted using a simple theoretical model of stress fiber dynamics and mechanics, supports the hypothesis that stretch-induced actin cytoskeletal remodeling and mechanotransduction via the MAPK pathways are regulated by perturbations in stress fiber tension. Importantly, the model illustrates a mechanism by which stress fibers respond to perturbations in cytoskeletal tension through tension relaxation and stress fiber reorientation to re-establish tensional homeostasis. Given that cyclic stretch induces perpendicular alignment and MAPK activation in many cell types, we expect that the results of this study to be of general relevance to many adherent cells. We wish to emphasize, however, that the model used herein describes general characteristics of stretch-induced stress fiber dynamics and does not attempt to describe finer details of cytoskeletal remodeling or intracellular signaling. Further, the role of cytoskeletal remodeling in stretch-induced activation of other intracellular signaling pathways and their functional consequences will need to be determined in future studies. Nonetheless, the results of this study indicate that this model provides a useful framework for exploring the mechanisms by which cytoskeletal tension and remodeling modulate stretch-induced mechanotransduction.

## Supporting Information

Text S1 Stochastic model of stretch-induced stress fiber reorganization. Contains the C++ code used to solve the model and sample output for the case of 10% cyclic uniaxial stretch at 1Hz.
(0.09 MB DOC)

Video S1 Cyclic equibiaxial stretch at 1Hz induces cell retraction. U2OS cells stably expressing GFP-actin subjected to 10% equibiaxial stretch at 1Hz. Cell retraction occurs within 5 minutes of starting cyclic stretch.
(1.91 MB MOV)

Video S2 Cyclic equibiaxial stretch at 0.01Hz does not induce a significant cell response. U2OS cells stably expressing GFP-actin subjected to 10% equibiaxial stretch at 0.01Hz. No significant cell retraction occurs after starting cyclic stretch.
(0.92 MB MOV)

Video S3 Step equibiaxial stretch does not induce a significant cell response. U2OS cells stably expressing GFP-actin do not show a significant changes in stress fiber dynamics in response to 10% equibiaxial stretch at t = 0. Bar = 20µm.
(1.12 MB MOV)

Video S4 Step equibiaxial stretch and release does not induce a significant cell response. U2OS cells stably expressing GFP-actin do not show a significant changes in stress fiber dynamics in response to releasing 10% equibiaxial stretch. The cells were stretched for 4 hours before releasing the stretch at t = 0. Bar = 20µm.
(1.14 MB MOV)
