# Supplementary material for: Stretch-Induced Stress Fiber Remodeling and the Activations of JNK and ERK Depend on Mechanical Strain Rate, but Not FAK
Source: PLoS One. 2010 Aug 30;5(8):e12470. doi: 10.1371/journal.pone.0012470 (PMC2930005; doi:10.1371/journal.pone.0012470)
Supplement: Text S1 — Stochastic model of stretch-induced stress fiber reorganization. Contains the C++ code used to solve the model and sample output for the case of 10% cyclic uniaxial stretch at 1Hz. (0.09 MB DOC) [file pone.0012470.s001.doc]

**C++ code and Sample Output for the Stochastic Model of Stretch-induced Stress Fiber Reorganization**

The system was solved using the program *stress_SF_model.cpp*, which was compiled and executed using the Microsoft Visual C++ compiler. The output data is saved in *file.txt* and *histogram.txt*. Since The program calls a random number generator (*rng_mt.cpp*) that chooses numbers between 0 and 1 with 53-bit resolution.

The numerical integrations were performed using a time increment Δ*t* of 0.01 sec for stretch frequencies at or below 1 Hz and 0.005 sec for frequencies between 1 and 10 Hz. The total number (*N*) of SFs in the simulations was 1000. In test cases, we found that increasing *N* reduced the noise in the output, but the system response was otherwise identical. Table 1 contains the input variables and the chosen values used in the present study. These values were determined by fitting the model to SF orientation distributions measured from bovine aortic endothelial cells subjected to 10% cyclic uniaxial stretch at frequencies of 0.01, 0.1 and 1 Hz [1].

**Table 1.** Variables and values used in *stress_SF_model.cpp* for 10% cyclic uniaxial stretch.

| **Variable** | **Value** | **Description** |
| --- | --- | --- |
| *Stretch* | 1.10 | the matrix stretch amplitude (10% stretch) |
| *Srdep* | 1.10 | *α_0_*; the stretch ratio of newly assembled SFs |
| *Srop* | 1.10 | *α_0_*; the set-point level of stretch |
| *k0* | 3.0 x10^-4^ | *k*_0_; from Eqn. S2 [s^-1^] |
| *k1* | 1.7x10^4^ | *k*_1_; from Eqn. S2 |
| *tvisc* | 0.5 | *τ*; from Eqn. S3 |
| *Fre* | 1.0 | *f*; the stretch frequency [s^-1^] |
| *SFnum* | 1000 | *N*; number of SFs |

***References***

1. Hsu HJ, Lee CF, Kaunas R (2009) A dynamic stochastic model of frequency-dependent stress fiber alignment induced by cyclic stretch. PLoS ONE 4: e4853.

***Sample Output***

*file.txt*

Each line of *file.txt* contains the following output variables for a single stretch cycle: time (seconds), the maximum and minimum values of average fiber stretch ratio$\left\langle\alpha^{i}\left(t\right)\right\rangle$, the maximum and minimum values of fiber tension$\left\langle f^{i}\left(t\right)\right\rangle$ (nN), the circular variance of fiber alignment, the order parameter *S*(*t*), and the standard deviation of *S*(*t*).

tvisc=0.5 k0=0.0003 k1=17000 frequency=1

time(sec) ASR(max) ASR(min) FiberTension(max) FiberTension(min) Circular_Variance average_cos Standard_Deviation

0 1.1 1.1 4.775 4.775 0.984901 -0.00937881 0.699353

1 1.13601 1.07891 6.60752 3.73818 0.985042 -0.0106089 0.699506

2 1.12685 1.07595 6.13408 3.59464 0.979871 -0.0128185 0.699504

. . . . . . . .

. . . . . . . .

. . . . . . . .

21598 1.10971 1.09062 5.26347 4.31228 0.367472 -0.63245 0.471684

21599 1.10971 1.0906 5.26347 4.31136 0.368426 -0.631516 0.472049

21600 1.10974 1.09059 5.26481 4.31115 0.368426 -0.631516 0.472049

*histogram.txt*

The file *histogram.txt* first records the orientations of the *i*th stress fiber (*i*=0 to 999) orientations at the 0, 0.5, 1, 4 and 6 hr time points. The values are summarized into histograms with bins of ‑90˚±7.5˚, -75˚±7.5˚, -60˚±7.5˚, -45˚±7.5˚, -30˚±7.5˚, -15˚±7.5˚, -0˚±7.5˚, 15˚±7.5˚, 30˚±7.5˚, 45˚±7.5˚, 60˚±7.5˚, and 75˚±7.5˚ for each time point.

tvisc=0.5 k0=0.0003 k1=17000 frequency=1

Repeat i time(hr) angle(radians)

0 0 0 0.780902

0 0 0.5 0.842821

0 0 1 -1.45196

0 0 4 1.15046

0 0 6 1.47413

0 1 0 0.699235

0 1 0.5 -1.05375

0 1 1 0.389173

0 1 4 1.00687

0 1 6 0.85691

. . . .

. . . .

. . . .

0 999 0 -0.610726

0 999 0.5 0.877239

0 999 1 0.877239

0 999 4 -1.37124

0 999 6 -0.78603

SFangle 0h 0.5h 1h 4h 6h

angle-90 76 186 211 227 219

angle-75 79 179 202 188 203

angle-60 100 117 120 107 101

angle-45 81 64 46 39 49

angle-30 82 35 28 23 25

angle-15 83 9 13 7 12

angle0 82 13 16 11 8

angle15 74 16 13 13 11

angle30 84 27 24 17 22

angle45 91 61 38 42 33

angle60 75 123 107 104 122

angle75 93 170 182 222 195

***The stochastic model of stretch-induced SF reorganization* (stress_SF_model.cpp)**

#include<fstream>

#include<stdio.h>

#include<stdlib.h>

#include<math.h>

#include<ctime>

#include <cstdio>

#include <cmath>

#define PI 3.141592653589793

using namespace std;

void init_genrand(unsigned long s);

void init_by_array(unsigned long init_key[], unsigned long key_length);

unsigned long genrand_int32(void);

long genrand_int31(void);

double genrand_real1(void);

double genrand_real2(void);

double genrand_real3(void);

double genrand_res53(void);

long double square(long double x);

int main(int argc, char *argv[]){

long double q[1000][2], x[2][1], theta[1][1000];

long double dfgrd[2][2], Histogram[1][1000][5];

long double h[5] = {0.0, 0.5, 1.0, 4.0, 6.0};

int SFangle[12][5] = {0};

int i, j, m, n2, h1, MassTurnover, OutputNo, cycleper100int;

int cycle, cycleper100sec;

double turnover_rate[1], CircularVariance[1];

long double freq = 1.0;

long double sr, srv, ASRmax, ASRmin;

long double FiberStrain, FiberTension, FTmax, FTmin;

long double k, p, probability, z;

long double str_inc, stretch = 1.1, srop = 1.1, srdep = 1.1;

long double etacos, etasin, Standard_Deviation;

long double k1 = 17000, k0 = 0.0003, SFnum = 1000.0, tvisc = 0.5;

long double poisson = 0.0;

long double dt = 1.0/freq/100, t, steps = 0.5/freq/dt;

long double str_t1, str_t2, cyclenum = 6.0*3600*freq;

ofstream outfile;

outfile.open("file.txt");

srand( (unsigned)time(0) );

unsigned long init[4] = {0x123, 0x234, 0x345, 0x456}, length = 4;

init_by_array(init, length);

cycleper100sec = 100 * freq;

cycleper100int = cycleper100sec;

outfile<<"tvisc="<<tvisc<<" "<<"k0="<<k0<<" "<<"k1="<<k1<<"

"<<"freqency="<<freq<<endl;

// Simulations //

for(j = 0; j < 1; j++){

OutputNo = 0;

etacos = 0.0;

etasin = 0.0;

MassTurnover = 0.0;

t = 0.0;

sr = 0.0;

FiberTension = 0.0;

Standard_Deviation = 0.0;

for(i = 0; i < SFnum ; i++){

theta[j][i] = PI * genrand_real1();

// theta is a random function between 0 and PI //

q[i][0] = srdep * cos(theta[j][i]);

q[i][1] = srdep * sin(theta[j][i]);

z = atan(q[i][1]/q[i][0]);

etacos = etacos + cos(2.0*z);

etasin = etasin + sin(2.0*z);

sr = sr + sqrt(square(q[i][0]) + square(q[i][1]));

FiberStrain = sqrt(square(q[i][0]) + square(q[i][1])) - 1;

FiberTension = FiberTension + 20.5*square(FiberStrain) +

45.7*FiberStrain;

// outfile<<i<<" "<<theta[j][i]<<endl;

if(theta[j][i] > PI){

printf("theta error\n");

}

}

for(i = 0; i < SFnum ; i++){

z = atan(q[i][1]/q[i][0]);

Standard_Deviation = Standard_Deviation +

square(cos(2.0*z)-etacos/SFnum);

}

Standard_Deviation = sqrt(Standard_Deviation/(SFnum-1));

sr = sr/SFnum;

FiberTension = FiberTension/SFnum;

ASRmax = sr;

ASRmin = sr;

FTmax = FiberTension;

FTmin = FiberTension;

CircularVariance[j] = 1.0 –

sqrt(square(etacos)+square(etasin))/SFnum;

printf("etacos = %15.12f, etasin = %15.12f, CircularVariance =

%15.12f \n", etacos, etasin, CircularVariance[j]);

outfile<<"time(sec)"<<" "<<"ASR(max)"<<" "<<"ASR(min)"<<"

"<<"FiberTension(max)"<<" "<<"FiberTension(min)"<<"

"<<"Circular_Variance"<<" "<<"average_cos"<<"

"<<"Standard_Deviation"<<endl;

outfile<<t<<" "<<ASRmax<<" "<<ASRmin<<" "<<FTmax<<" "<<FTmin<<"

"<<CircularVariance[j]<<" "<<etacos/SFnum<<"

"<<Standard_Deviation<<endl; // write to file.txt //

for(cycle = 1; cycle <= cyclenum; cycle++){

ASRmax = 0.0;

ASRmin = stretch * srop;

FTmax = 0.0;

FTmin = 20.5*square(stretch*srop-1) +

45.7*(stretch*srop-1);

// create n steps of stretch //

str_t1 = srdep;

for(n2 = 1; n2 <= steps; n2++){

t = t + dt;

str_t2 = (srdep * (stretch-1.0)/2.0) *

(sin(2*PI*freq*t - PI/2.0)+ 1.0 ) + 1.1;

str_inc = str_t2 / str_t1;

str_t1 = str_t2;

// create greens deformation gradient tensor for step

stretch //

dfgrd[0][0] = str_inc;

dfgrd[0][1] = 0.0;

dfgrd[1][0] = 0.0;

dfgrd[1][1] = 1.0 - poisson*(str_inc-1.0);

// stretch all fibers one increment

for(i = 0; i < SFnum; i++){

// calculate axial strain ratio in stretched

fibers

x[0][0] = dfgrd[0][0]*q[i][0] +

dfgrd[0][1]*q[i][1];

x[1][0] = dfgrd[1][0]*q[i][0] +

dfgrd[1][1]*q[i][1];

sr = sqrt(square(x[0][0]) + square(x[1][0]));

srv = srop + (sr - srop) * exp(-dt/tvisc);

x[0][0] = x[0][0]*srv/sr;

x[1][0] = x[1][0]*srv/sr;

k = k0 * (1 + k1 * square((srv-srop)/srop));

q[i][0] = x[0][0];

q[i][1] = x[1][0];

p = genrand_res53();

probability = k*dt;

if(p>1.0){

printf("p error\n");

}

if (p <= probability) {

theta[j][i] = genrand_real1();

theta[j][i] = PI*theta[j][i];

q[i][0] = srdep * cos(theta[j][i]);

q[i][1] = srdep * sin(theta[j][i]);

MassTurnover = MassTurnover + 1;

if(theta[j][i] > PI){

printf("theta error\n");

}

}

if (cycle == 6.0 * 3600 * freq){

z = atan(q[i][1]/q[i][0]);

Histogram[j][i][4] = z;

}

if (cycle == 4.0 * 3600 * freq){

z = atan(q[i][1]/q[i][0]);

Histogram[j][i][3] = z;

}

if (cycle == 1.0 * 3600 * freq){

z = atan(q[i][1]/q[i][0]);

Histogram[j][i][2] = z;

}

if (cycle == 0.5 * 3600 * freq){

z = atan(q[i][1]/q[i][0]);

Histogram[j][i][1] = z;

}

if (cycle == 1){

z = atan(q[i][1]/q[i][0]);

Histogram[j][i][0] = z;

}

}

sr = 0.0;

FiberTension = 0.0;

for(i = 0; i < SFnum; i++){

sr = sr + sqrt(square(q[i][0]) +

square(q[i][1]));

FiberStrain = sqrt(square(q[i][0]) + square(q[i][1]))

- 1;

FiberTension = FiberTension +

20.5*square(FiberStrain) + 45.7*FiberStrain;

}

sr = sr/SFnum;

if(sr > ASRmax){

ASRmax = sr;

}

else{

ASRmax = ASRmax;

}

if(sr < ASRmin){

ASRmin = sr;

}

else{

ASRmin = ASRmin;

}

FiberTension = FiberTension/SFnum;

if(FiberTension > FTmax){

FTmax = FiberTension;

}

else{

FTmax = FTmax;

}

if(FiberTension < FTmin){

FTmin = FiberTension;

}

else{

FTmin = FTmin;

}

}

// create n steps of release //

str_t1 = srdep*stretch;

for(n2 = 1; n2 <= steps; n2++){

t = t + dt;

str_t2 = (srdep * (stretch-1.0)/2.0) *

(sin(2*PI*freq*t - PI/2.0) + 1.0) + 1.1;

str_inc = str_t2/str_t1;

str_t1 = str_t2;

// create greens deformation gradient tensor for step

release //

dfgrd[0][0] = str_inc;

dfgrd[0][1] = 0.0;

dfgrd[1][0] = 0.0;

dfgrd[1][1] = 1.0 - poisson*(str_inc-1.0);

// stretch all fibers one increment

for(i = 0; i < SFnum; i++){

// calculate axial strain ratio in stretched

fibers

x[0][0] = dfgrd[0][0]*q[i][0] +

dfgrd[0][1]*q[i][1];

x[1][0] = dfgrd[1][0]*q[i][0] +

dfgrd[1][1]*q[i][1];

sr = sqrt(square(x[0][0])+square(x[1][0]));

srv = srop + (sr - srop)*exp(-dt/tvisc);

x[0][0] = x[0][0]*srv/sr;

x[1][0] = x[1][0]*srv/sr;

k = k0 * (1 + k1 * square((srv-srop)/srop));

q[i][0] = x[0][0];

q[i][1] = x[1][0];

p = genrand_res53();

probability = k*dt;

if(p>1.0){

printf("p error\n");

}

if (p <= probability){

theta[j][i] = genrand_real1();

theta[j][i] = PI*theta[j][i];

q[i][0] = srdep*cos(theta[j][i]);

q[i][1] = srdep*sin(theta[j][i]);

MassTurnover = MassTurnover + 1;

if(theta[j][i]>PI){

printf("theta error\n");

}

}

if (cycle == 6.0*3600*freq){

z = atan(q[i][1]/q[i][0]);

Histogram[j][i][4] = z;

}

if (cycle == 4.0*3600*freq){

z = atan(q[i][1]/q[i][0]);

Histogram[j][i][3] = z;

}

if (cycle == 1.0 * 3600 * freq){

z = atan(q[i][1]/q[i][0]);

Histogram[j][i][2] = z;

}

if (cycle == 0.5 * 3600 * freq){

z = atan(q[i][1]/q[i][0]);

Histogram[j][i][1] = z;

}

if (cycle == 1 ){

z = atan(q[i][1]/q[i][0]);

Histogram[j][i][0] = z;

}

}

sr = 0.0;

FiberTension = 0.0;

for(i = 0; i < SFnum; i++){

sr = sr + sqrt(square(q[i][0]) +

square(q[i][1]));

FiberStrain = sqrt(square(q[i][0]) +

square(q[i][1])) - 1;

FiberTension = FiberTension +

20.5*square(FiberStrain) + 45.7*FiberStrain;

}

sr = sr/SFnum;

if(sr > ASRmax){

ASRmax = sr;

}

else{

ASRmax = ASRmax;

}

if(sr < ASRmin){

ASRmin = sr;

}

else{

ASRmin = ASRmin;

}

FiberTension = FiberTension/SFnum;

if(FiberTension > FTmax){

FTmax = FiberTension;

}

else{

FTmax = FTmax;

}

if(FiberTension < FTmin){

FTmin = FiberTension;

}

else{

FTmin = FTmin;

}

}

etacos = 0.0;

etasin = 0.0;

for(i = 0;i<SFnum;i++){

z = atan(q[i][1]/q[i][0]);

etacos = etacos + cos(2.0*z);

etasin = etasin + sin(2.0*z);

}

Standard_Deviation = 0.0;

for(i = 0; i < SFnum ; i++){

z = atan(q[i][1]/q[i][0]);

Standard_Deviation = Standard_Deviation +

square(cos(2.0*z)-etacos/SFnum);

}

Standard_Deviation = sqrt(Standard_Deviation/(SFnum-1));

// record data for every cycle

OutputNo = OutputNo + 1;

CircularVariance[j] = 1.0 - sqrt(square(etacos) +

square(etasin))/SFnum;

//printf("cycle: %d, ASRmax: %f, ASRmin: %f,

CircularVariance: %f \n",cycle, ASRmax, ASRmin,

CircularVariance[j]);

outfile<<t<<" "<<ASRmax<<" "<<ASRmin<<" "<<FTmax<<"

"<<FTmin<<" "<<CircularVariance[j]<<"

"<<etacos/SFnum<<" "<<Standard_Deviation<<endl;

// write to file.txt //

}

}

outfile.close(); // close file.text //

// open histogram.txt file to record the orientation of stress fibers //

ofstream myfile("histogram.txt");

myfile.is_open();

for(j = 0; j<1; j++){

// write to histogram.txt //

myfile<<"tvisc="<<tvisc<<" "<<"k0="<<k0<<" "<<"k1="<<k1<<"

"<<"frequency="<<freq<<endl;

myfile<<"Repeat"<<" "<<"SFnum"<<" "<<"hour"<<"

"<<"angle"<<endl;

for(i = 0; i < SFnum; i++){

for (h1 = 0; h1 < 5; h1++){

myfile<<j<<" "<<i<<" "<<h[h1]<<"

"<<Histogram[j][i][h1]<<endl;

// write to histogram.txt //

if(Histogram[j][i][h1]*180/PI >= -90.0 &&

Histogram[j][i][h1]*180/PI < -82.5){

SFangle[0][h1]++;

}

else if(Histogram[j][i][h1]*180/PI >= -82.5 &&

Histogram[j][i][h1]*180/PI < -67.5){

SFangle[1][h1]++;

}

else if(Histogram[j][i][h1]*180/PI >= -67.5 &&

Histogram[j][i][h1]*180/PI < -52.5){

SFangle[2][h1]++;

}

else if(Histogram[j][i][h1]*180/PI >= -52.5 &&

Histogram[j][i][h1]*180/PI < -37.5){

SFangle[3][h1]++;

}

else if(Histogram[j][i][h1]*180/PI >= -37.5 &&

Histogram[j][i][h1]*180/PI < -22.5){

SFangle[4][h1]++;

}

else if(Histogram[j][i][h1]*180/PI >= -22.5 &&

Histogram[j][i][h1]*180/PI < -7.5){

SFangle[5][h1]++;

}

else if(Histogram[j][i][h1]*180/PI >= -7.5 &&

Histogram[j][i][h1]*180/PI < 7.5){

SFangle[6][h1]++;

}

else if(Histogram[j][i][h1]*180/PI >= 7.5 &&

Histogram[j][i][h1]*180/PI < 22.5){

SFangle[7][h1]++;

}

else if(Histogram[j][i][h1]*180/PI >= 22.5 &&

Histogram[j][i][h1]*180/PI < 37.5){

SFangle[8][h1]++;

}

else if(Histogram[j][i][h1]*180/PI >= 37.5 &&

Histogram[j][i][h1]*180/PI < 52.5){

SFangle[9][h1]++;

}

else if(Histogram[j][i][h1]*180/PI >= 52.5 &&

Histogram[j][i][h1]*180/PI < 67.5){

SFangle[10][h1]++;

}

else if(Histogram[j][i][h1]*180/PI >= 67.5 &&

Histogram[j][i][h1]*180/PI < 82.5){

SFangle[11][h1]++;

}

else{

SFangle[0][h1]++;

}

}

}

}

// write to histogram.txt //

myfile<<"SFangle"<<" "<<"0h"<<" "<<"0.5h"<<" "<<"1h"<<" "<<"4h"<<"

"<<"6h"<<" "<<endl;

for(m = 0; m < 12; m++){

myfile<<"angle"<<-90+15*m<<" "<<SFangle[m][0]<<"

"<<SFangle[m][1]<<" "<<SFangle[m][2]<<"

"<<SFangle[m][3]<<" "<<SFangle[m][4]<<endl;

}

myfile.close(); // close histogram.txt file //

return EXIT_SUCCESS;

}

// create Square function //

long double square(long double x){

long double y;

y = (x)*(x);

return y;

}

***Random Number Generator* (rng_mt.cpp)**

#include <cstdio>

using namespace std;

/* Period parameters */

#define N 624

#define M 397

#define MATRIX_A 0x9908b0dfUL /* constant vector a */

#define UPPER_MASK 0x80000000UL /* most significant w-r bits */

#define LOWER_MASK 0x7fffffffUL /* least significant r bits */

static unsigned long mt[N]; /* the array for the state vector */

static int mti=N+1; /* mti==N+1 means mt[N] is not initialized */

/* initializes mt[N] with a seed */

void init_genrand(unsigned long s)

{

mt[0]= s & 0xffffffffUL;

for (mti=1; mti<N; mti++) {

mt[mti] = (1812433253UL * (mt[mti-1] ^ (mt[mti-1] >> 30)) + mti);

/* See Knuth TAOCP Vol2. 3rd Ed. P.106 for multiplier. */

/* In the previous versions, MSBs of the seed affect */

/* only MSBs of the array mt[]. */

/* 2002/01/09 modified by Makoto Matsumoto */

mt[mti] &= 0xffffffffUL;

/* for >32 bit machines */

}

}

/* initialize by an array with array-length */

/* init_key is the array for initializing keys */

/* key_length is its length */

//void init_by_array(init_key, key_length)

//unsigned long init_key[], key_length;

void init_by_array(unsigned long init_key[], unsigned long key_length)

{

int i, j, k;

init_genrand(19650218UL);

i=1; j=0;

k = (N>key_length ? N : key_length);

for (; k; k--) {

mt[i] = (mt[i] ^ ((mt[i-1] ^ (mt[i-1] >> 30)) * 1664525UL)) + init_key[j] + j; /* non linear */

mt[i] &= 0xffffffffUL; /* for WORDSIZE > 32 machines */

i++; j++;

if (i>=N) { mt[0] = mt[N-1]; i=1; }

if (j>=key_length) j=0;

}

for (k=N-1; k; k--) {

mt[i] = (mt[i] ^ ((mt[i-1] ^ (mt[i-1] >> 30)) * 1566083941UL)) - i; /* non linear */

mt[i] &= 0xffffffffUL; /* for WORDSIZE > 32 machines */

i++;

if (i>=N) { mt[0] = mt[N-1]; i=1; }

}

mt[0] = 0x80000000UL; /* MSB is 1; assuring non-zero initial array */

}

/* generates a random number on [0,0xffffffff]-interval */

unsigned long genrand_int32(void)

{

unsigned long y;

static unsigned long mag01[2]={0x0UL, MATRIX_A};

/* mag01[x] = x * MATRIX_A for x=0,1 */

if (mti >= N) { /* generate N words at one time */

int kk;

if (mti == N+1) /* if init_genrand() has not been called, */

init_genrand(5489UL); /* a default initial seed is used */

for (kk=0;kk<N-M;kk++) {

y = (mt[kk]&UPPER_MASK)|(mt[kk+1]&LOWER_MASK);

mt[kk] = mt[kk+M] ^ (y >> 1) ^ mag01[y & 0x1UL];

}

for (;kk<N-1;kk++) {

y = (mt[kk]&UPPER_MASK)|(mt[kk+1]&LOWER_MASK);

mt[kk] = mt[kk+(M-N)] ^ (y >> 1) ^ mag01[y & 0x1UL];

}

y = (mt[N-1]&UPPER_MASK)|(mt[0]&LOWER_MASK);

mt[N-1] = mt[M-1] ^ (y >> 1) ^ mag01[y & 0x1UL];

mti = 0;

}

y = mt[mti++];

/* Tempering */

y ^= (y >> 11);

y ^= (y << 7) & 0x9d2c5680UL;

y ^= (y << 15) & 0xefc60000UL;

y ^= (y >> 18);

return y;

}

/* generates a random number on [0,0x7fffffff]-interval */

long genrand_int31(void)

{

return (long)(genrand_int32()>>1);

}

/* generates a random number on [0,1]-real-interval */

double genrand_real1(void)

{

return genrand_int32()*(1.0/4294967295.0);

/* divided by 2^32-1 */

}

/* generates a random number on [0,1)-real-interval */

double genrand_real2(void)

{

return genrand_int32()*(1.0/4294967296.0);

/* divided by 2^32 */

}

/* generates a random number on (0,1)-real-interval */

double genrand_real3(void)

{

return (((double)genrand_int32()) + 0.5)*(1.0/4294967296.0);

/* divided by 2^32 */

}

/* generates a random number on [0,1) with 53-bit resolution*/

double genrand_res53(void)

{

unsigned long a=genrand_int32()>>5, b=genrand_int32()>>6;

return(a*67108864.0+b)*(1.0/9007199254740992.0);

}

/* These real versions are due to Isaku Wada, 2002/01/09 added
